# A diffusion based study of population dynamics: Prehistoric migrations into South Asia

**DOI:** 10.1371/journal.pone.0176985

**Published:** 2017-05-11

**Authors:** Mayank N. Vahia, Nisha Yadav, Uma Ladiwala, Deepak Mathur

**Affiliations:** Tata Institute of Fundamental Research, Mumbai, India; Harvard Medical School, UNITED STATES

## Abstract

A diffusion equation has been used to study migration of early humans into the South Asian subcontinent. The diffusion equation is tempered by a set of parameters that account for geographical features like proximity to water resources, altitude, and flatness of land. The ensuing diffusion of populations is followed in time-dependent computer simulations carried out over a period of 10,000 YBP. The geographical parameters are determined from readily-available satellite data. The results of our computer simulations are compared to recent genetic data so as to better correlate the migratory patterns of various populations; they suggest that the initial populations started to coalesce around 4,000 YBP before the commencement of a period of relative geographical isolation of each population group. The period during which coalescence of populations occurred appears consistent with the established timeline associated with the Harappan civilization and also, with genetic admixing that recent genetic mapping data reveal. Our results may contribute to providing a timeline for the movement of prehistoric people. Most significantly, our results appear to suggest that the Ancestral Austro-Asiatic population entered the subcontinent through an easterly direction, potentially resolving a hitherto-contentious issue.

## Introduction

The primary drivers for prehistoric human migrations have been geography and availability of natural resources [1]. These are deterministic parameters when small populations relocate to newer territories under conditions in which conflicts and large group dynamics are inconsequential. In order to understand the dynamics of the movement of prehistoric people, we have developed a computer simulation code based on habitability, which we define in terms of the altitude of a given geographical location, its proximity to water resources, and the flatness of land; these parameters are utilized to temper a diffusion equation that helps determine the preferred path of migration [2]. We make use of high resolution topographical and hydro-shed data from readily accessible satellite databases to define these habitability parameters. This enables us to simulate the diffusion of populations based on human preference for more habitable places, with the rate of movement tempered by existing populations. We check the veracity of the results of our computer simulations by making comparisons with genetic data. Our simulation is seen to predict fairly accurately the points of contact between different migratory paths. Such comparison also opens possibilities for developing fresh insights into both the path and the temporal dynamics of the movement of people over ~10,000 years before the present era.

India, well-known for the enormous diversity of its 1.2 billion human population, has been a major corridor for prehistoric migrations. It has been estimated that, currently, the country harbours more than 4,600 ethnic groups [3]. The diversity within the overall population manifests itself in language as well as in genetic make-up. The social structure has resulted from stratification of the Indian population into castes and tribes, with the prevailing semi-feudal character of the subcontinent ensuring that these usually rigid social classifications continue despite urbanisation and concomitant societal changes.

The origins of these populations are embedded in the fact that India has been an important conduit for several migrations since prehistory. Indeed, the peopling of India occurred as a consequence of one of the early waves of migration out of Africa [4–6]. However, it remains a subject of controversy whether this out-of-Africa migration occurred in one or several waves [7, 8]. One commonly accepted hypothesis is that Homo Sapiens came to the Indian Subcontinent in three major waves [9]. The earliest Paleolithic migration has been postulated to have taken place 60,000–40,000 years before present (YBP) [10]. This migration involved a southern exit from Africa, along a coastal route from the Middle East to India, and then to South East Asia [10]. A later migration occurred around 45,000 YBP from the North West, across Central Asia into North-western India. India also has a significant group with genetic affinity to the Tibetan and Burmese (Myanmar) population [9]. Possehl [11] has discussed the possible routes taken by incoming populations and it is surmised that the group from Central Asia, while entering India split into two, one going to northern Tibet and the other to the Eastern part of the subcontinent. The simplest path seems to be through Khyber Pass, along the foothills of the Himalayas into the Brahmaputra plains. An alternate path from Tibet via Brahmaputra itself is possible but may, perhaps, be more difficult than the one along the foothills of Himalayas.

The mainland Indian population has, in the literature, been broadly divided in one of the two ways: either culturally into castes and tribes, or linguistically into four major, almost non-overlapping families—the Dravidian (DR) speaking groups inhabit southern India, the Indo-European (IE) speaking groups inhabit northern India, Tibeto-Burman (TB) speaking groups inhabit north-eastern India and the relatively sparse Austro-Asiatic (AA) speakers are found in tribes of central and eastern India. There is an additional distinct group found in the Andaman Islands who are believed to be descendants of the early Austro-Asiatic who invaded the islands from East Asia [12]. The ancestral populations of these linguistic groups are correspondingly, the Ancestral South Indian (ASI), Ancestral North Indian (ANI), Ancestral Tibeto-Burman (ATB) and the Ancestral Austro-Asiatic (AAA). According to one viewpoint, the ASI are considered an offshoot of the AAA [5].

**Fig 1:**
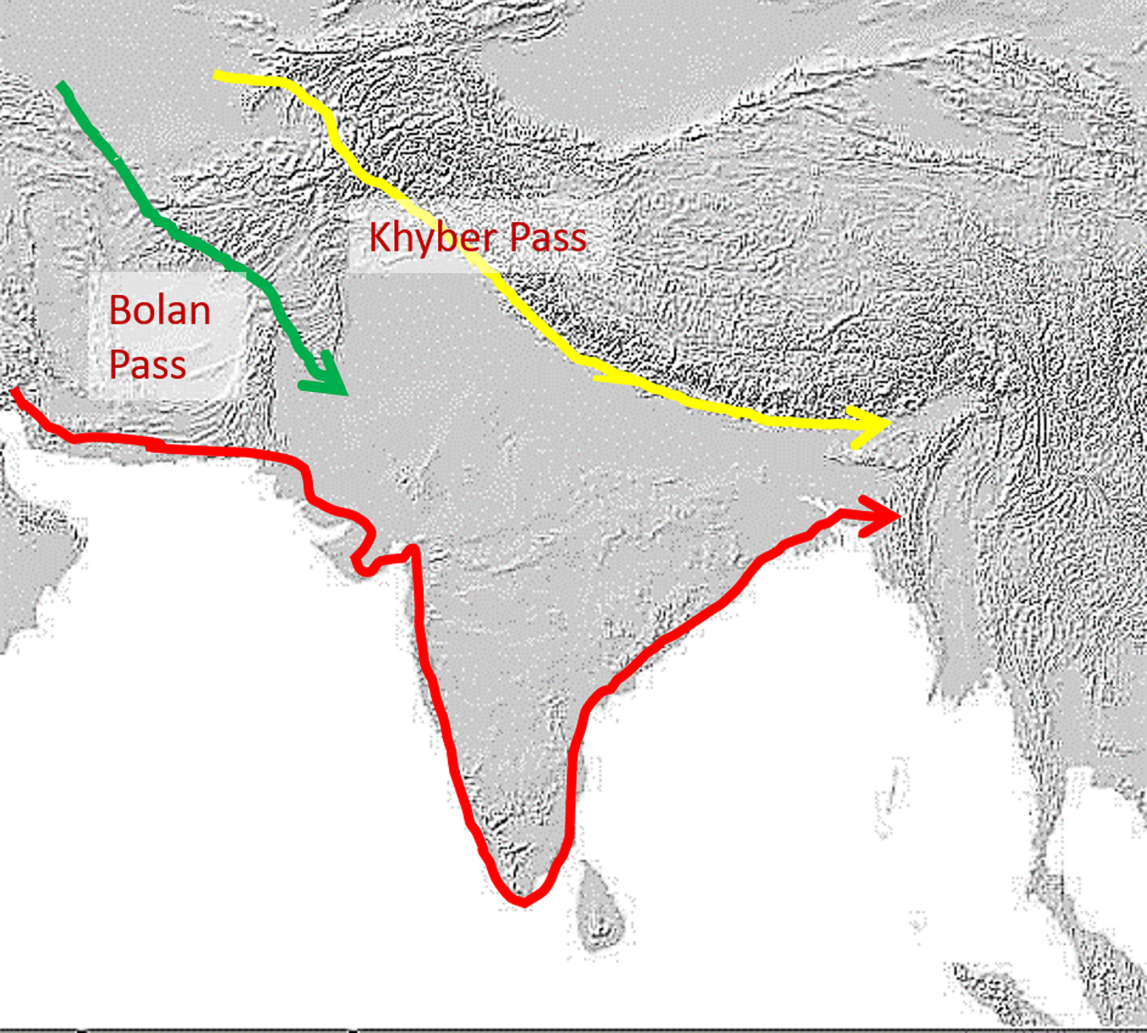
Potential migration routes. Some potential migration routes of various population groups entering the South Asian subcontinent more than 30,000 years ago. Red indicates the path of the earliest migrants along the coastal route; Yellow denotes entrants from the Khyber Pass and green indicates the path taken by migrants from Iran via the Bolan Pass.

Genetic studies of the Indian population have been few and recent. Evidence from earlier studies seems to imply that the majority of the population was descended from varying mixtures of the ANI and ASI [13]. A more recent genome-based study found ancestral contributions by the AAA and ATB, in addition to the ANI and ASI [3, 14]. Some potential entry routes of major groups that had occupied the subcontinent by 30,000 YBP are shown in Fig 1 [15].

In addition to these two divergent perspectives on the ancestry of the Indian population, other important facets continue to remain unclear. Most important amongst contemporary concerns are the movement of people within the subcontinent. It would appear safe to assume that upon initial arrival into the subcontinent—which was extremely rich in natural resources [16, 17]—the initial migrants would have tended to remain geographically localized. The need for further migrations could then have been stimulated by one or more of the following conditions:

1. Build-up of significant population pressure in a given location;
2. Internal strife;
3. Climatic changes;
4. Identification of new resource-rich sites by occasional wanderers/explorers.

The possible influence of climatic changes on these conditions continues to be a subject of considerable debate (see, for instance, [18, 19] and references therein). However, there seems to be consensus that climatic conditions exert an influence on the richness of resources that would, in turn, affect the habitability and desirability of particular geographical locations. The most important climatic changes that affect resource richness include large-scale eruptions of volcanoes, glacial events, alterations in monsoon patterns and sea-level fluctuations [20–23]; we expect these to exert minimal influence on population dynamics on the 10,000 year timescales that we are considering in this study. The starting time of 10,000 YBP for our simulations was selected as it essentially marks the beginning of the Holocene period; it also coincides with a substantial rise in mean sea level (as much as 60 m, as shown in [24], and rise in monsoon activity in the Indian subcontinent [25]). Field et al. [26] have explicitly considered the possible effects on population dynamics of rise in sea levels that occurred 12,000 YBP. Their studies imply that any effects would have impacted mostly the coastal corridors, perhaps leading to a hastening of population dispersal into the heartlands of the subcontinent. The coastal corridor along the Western coast of the subcontinent has been estimated to be about 100 km wide [27].

We have attempted to gain insights into how the Indian subcontinent was populated in prehistoric times by adopting an entirely different (non-linguistic, non-caste/tribe) approach. We have employed a diffusion model to study migration of early humans into the Indian subcontinent. Such a diffusion-based technique has, recently, been successfully adapted by us to study prehistoric migration into the main British island [2], with our results being validated by available genetic data. The British island comprising present-day England, Scotland and Wales presented to us a geographically isolated system. On the other hand, our present study of the Indian subcontinent presents a system that is very well connected, geographically, to vast neighbouring locations. Nevertheless, as before, the population dynamics in our present study are also governed by a set of parameters that account for geographical features such as proximity to water resources, altitude, and flatness of land. These factors temper the diffusion of populations which we follow in time-dependent computer simulations carried out over a period of 10,000 YBP.

Our population dynamics simulation code is based on human affinity to habitable land. The habitability of land is defined in terms of parameters like availability of water sources, altitude, and flatness of land. These parameters act as determinants of migration, as discussed in the following section. We simulate the prehistoric movement of people from initial entry points that we identify on the basis of data from the paleolithic period. We use current topography and hydro-shed information from satellite-based data on the Earth’s topography to define habitability using the above parameters. We then simulate the movement of populations assuming that they have an affinity to move towards more habitable places, with the rate of movement tempered by existing populations. We make quantitative comparison of the results of our computer simulations with genetic data that has, in recent years, become available for the Indian sub-continent. Specifically, we make comparison of our results with genetic data in those geographical locations where distinct population groups merge. Our results appear to suggest that such mergers occur most prominently in central India. These locations are expected to display a mix of different genetic groups, with less likelihood of a single group dominating. Therefore, by making our comparisons in regions of population merger, we seek to avoid potential saturation effects from manifesting themselves. Our comparison indicates that our simulations seem to be able to predict the points of contacts between different groups and their mutual influence and, also, yield information about the path of peoples’ movement over a period of ~10,000 years before the present era.

## Methods

### Computer simulations of population dynamics

Availability of ancient geographical data is a prerequisite for carrying out prehistoric population dynamics studies based on diffusion tempered by factors such as flatness of land and proximity to water bodies. Our present study has been carried out over a period of about 10,000 years before the present era; as timescales for geological changes are considerably longer than this, it is appropriate for us to make use of contemporary geographical data. In the present study we have made use of satellite maps of the Earth’s surface (GLOBE Digital Elevation Model from NOAA–the National Oceanic and Atmospheric Administration—http://www.ngdc.noaa.gov/mgg/topo/globe.html), which provides us the geographical data with 8 km resolution. We also rely on satellite data to obtain information on water resources, making use of high accuracy hydro-shed data (http://hydrosheds.cr.usgs.gov/dataavail.php). Higher resolution satellite data are, indeed, readily available (down to 30 m resolution) but we found it easier (from the perspective of computer time) to use the 8 km-resolution data for our computations, without adverse effect on overall accuracy. We have confirmed (by running simulations over limited geographical areas using high resolution data) that spatial distances of hundreds kilometres covered in our present simulations are adequately represented using 8 km resolution: 30 m resolution is gross overkill. The accompanying computation expense is may (possibly) be justified only if we were simulating over distances of the order of a few tens of kilometers.

Our diffusion model is based on solutions of the generalised Fisher equation:

$$\frac{\partial N}{\partial t}+(V•\nabla )N=\gamma N(1-\frac{N}{k})+\nabla •(\nu \nabla N),$$ (1)

where N represents population density, V the velocity of directed motion of people, γ is the rate at which population increases, k is the carrying capacity of the land and v is the diffusion coefficient. The time and spatial variation of the population is defined by the left hand side of the equation while the right hand side takes cognizance of the rate of population increase, the ability of the land to sustain the population, and the tendency of people to explore surrounding areas.

Availability of geographical data from prehistoric times may, *a priori*, be considered to be a prerequisite for carrying out our population dynamics studies. However, as the present study is over a period of ~10,000 years before the present era—and timescales for geological changes are considerably longer than this–we consider it appropriate to make use of contemporary geographical data as made available in satellite maps of the Earth’s surface (GLOBE Digital Elevation Model from NOAA–the National Oceanic and Atmospheric Administration [28] and information on water resources from high accuracy hydro-shed data [29].

In order to simulate the diffusive movement of the people, we divided the Indian subcontinent into square grids of 8 km x 8 km size. We placed initial populations in selected grids (as discussed below) and assumed that diffusion of people will result in movement to any of the adjoining grids on the basis of the following four parameters (for a detailed description of the simulation algorithm, see [2]):

1. Altitude;
2. Surface-kind–a parameter of relative flatness;
3. Proximity to water source;
4. Population density.

The veracity of these four parameters has been recently tested by us in a “test case” which involved the diffusional dynamics of prehistoric populations in the main British island [2]. Our results were in consonance with recent genetic mapping of England, Scotland and Wales.

In the list of four parameters, we relate Parameter 1, denoted D_alt_, to the integrated population density which, as has shown earlier [30], decreases faster than exponentially as the altitude increases from sea level to ~1000 m; thereafter, it slowly rises again so as to peak at ~2300 m before falling off again. A cut-off altitude occurs at ~4000 m. Parameter 2 (denoted D_surf_) differs from Parameter 1 in that it accounts for the *slope* of the land. We assume that, even if a given location is not at a high altitude, if it is sloping, it becomes less desirable. Of course humans have historically adapted to sloping land by cutting into, or flattening, a local portion; it is because of this that D_surf_ is relatively weakly dependent on the diffusion parameter. Parameter 3 (denoted D_alt_) is self-explanatory and is based on the work reported by Cohen et al. [30]. Parameter 4 is initially provided externally but a maximum value of population density is defined that seeks to take cognizance of the how much population density a region can sustain based on the values of the first three parameters.

In general terms, the population traversing out of or into a given location is decided by a rate for emigration and a rate for immigration, both of which are decided by the values of the four parameters described above. These, taken together, define the desirability of a region of land. If the population exceeds a certain fraction of the maximum sustainable population, our computer program significantly increases the emigration rate and blocks immigration until the population comes down significantly, below the maximum sustainable population. Values of Parameter 4 range from 0 to 1 for a land mass; we take it to be -1 for a water source. A linear combination of the four parameters is used determine the absolute rating of a given location, denoted R_in_, which largely depends on geographical parameters; it changes dynamically with a location’s population, and corresponds to habitability whenever the population is 0. Details of how values are assigned to each parameter are discussed in the following.

The suitability of a location is quantitatively described in terms of normalized parameters, listed above, and is called desirability. Thus, there is desirability that is based on: altitude, surface-flatness, and water-proximity.

#### Desirability based on altitude: D_alt_

**Fig 2:**
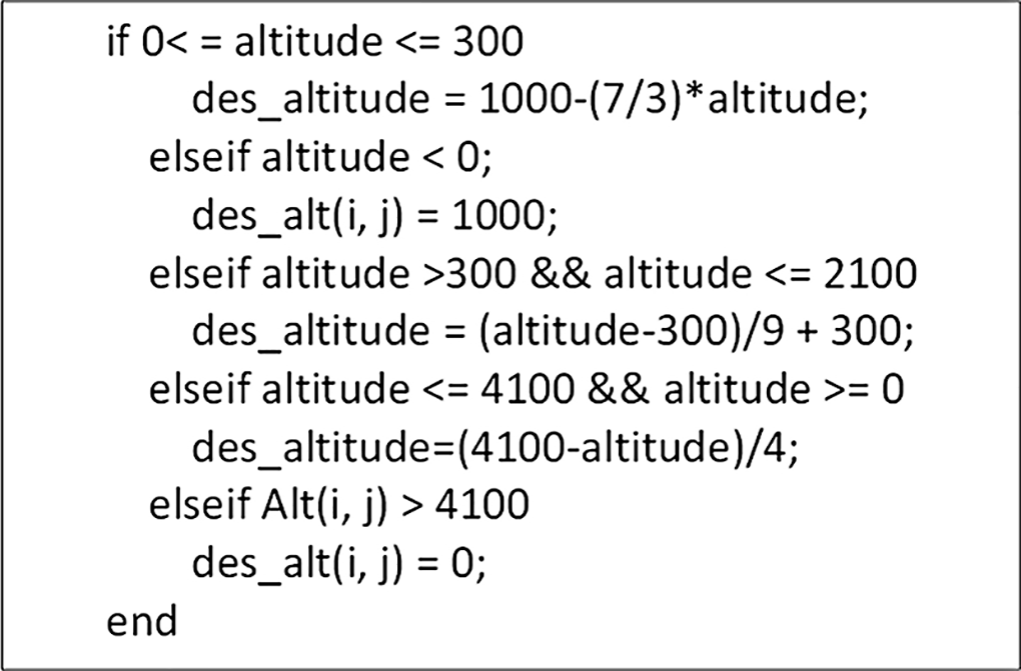
Algorithm used. The algorithm used for sensitivity of habitability on altitude.

D_alt_ (coded as des alt in Fig 2) is a piecewise defined linear function against altitude; its value is normalized to lie between 0 and 1. The algorithm is depicted in Fig 2.

#### Desirability based on surface kind: D_surf_

The mean altitude of a place (A_mean_) is calculated on the basis of the NASA satellite database. Three kinds of surfaces are considered by our program: flat, tolerable, ignore. The surface-kind depends on the deviation *d* of a location from the mean altitude of the region. If *d<*L_flat_ (nominally set at 0.25), the surface is designated as flat; the value of D_surf_ then falls off (from 1) in a power-law [30]. If L_tol_>*d>*L_flat_, the surface is designated tolerable; D_surf_ then falls off much more rapidly. Every other location is assumed to be uninhabitable and D_surf_ = 0.

#### Desirability based on location: D_loc_

D_loc_ is a linear combination of D_alt_ and D_surf_ with D_alt_ having weightage, W_flat_. Parameters W_flat_, L_tol_, L_flat_ are all variables that can be changed in the variables file in our program, keeping the original simulation file unaltered. The entire simulation is carried out in Matlab using code that is developed in-house.

#### Desirability based on water-source proximity

There are three different models

1. D_riv_ and distance to the water source follow an inverse square law [31]. We consider any water source that lies within 10 kilometres as acceptable.
2. D_riv_ and distance to the water source follow a linear square law [31] with the same range as above.
3. D_riv_ follows the same relations as (1) but has double the range (20 km).

#### Rating of each site R_in_

We calculate the population independent rating of each site, R_in_, as a linear combination of D_riv_ and D_loc_. For flat lands, 0.5<R_in_<1. For tolerable lands, 0.25<R_in_<1.

#### Optimum population of a grid

We calculate the optimum population that a location can sustain as the arithmetic mean of water-based optimum population and location-based optimum population. Both are a product of different parameters of desirability and P_max i_ for any location *i*.

#### Description of our simulation-engine

We pre-compute values of R_in_ and optimum population (P_best_) where the rating R_i_ of a location *i* is calculated as:

$$R_{i}=R_{in}+C*P_{i},$$ (2)

where C is a constant and P_i_ is the currently existing population. C is nominally taken as 1. In a given location, the rating-per-unit-population is deduced to be α for that place where α is a linear combination of various desirability parameters.

Along the x-axis and the y-axis of a Cartesian coordinate system depicting population dynamics, migrating people tend to move along the direction with greater mean of α. The fraction of people moving towards positive x direction is taken to be f_x_ and those moving towards the y direction is taken to be f_y_. For a given direction and a given boundary, the difference of α’s is taken to be δ. The polarization of migrating people towards each of the opposite boundaries is defined as the function of two δ’s and is called β, a parameter that is taken to be equivalent to the “pressure” felt by a boundary. The population moving towards a given boundary can be represented in terms of the fraction of people moving in (F_i_) and out (F_o_), is defined as

$$F_{i}=f*\beta$$ (3)

$$F_{0}=f*(1-\beta ).$$ (4)

Once these parameters are defined, our diffusion model is used with rating instead of pressure. We define an emigration rate E_i_ from a cell *i* given by N_i_*R_i_. In a given boundary, if N is the number of people moving towards the cell, the immigration rate (I_i_)—the number of people moving out–can be denoted as

$$I_{i}=\sum_{j}N*R_{i}/\sum_{j}R_{i}$$ (5)

for *j =* 4 cells north, south, east and west. Hence, for every boundary we have emigration and immigration calculated as Em, Im or Em1, Im1 (for rows and columns). The net flux at any given boundary is Em-Im. The change in a cell is the net flux across all four boundaries. We compute the change matrix so as to monitor patterns. Population increase due to net birth is defined as 0.001*P_i_*R_i_.

**Fig 3:**
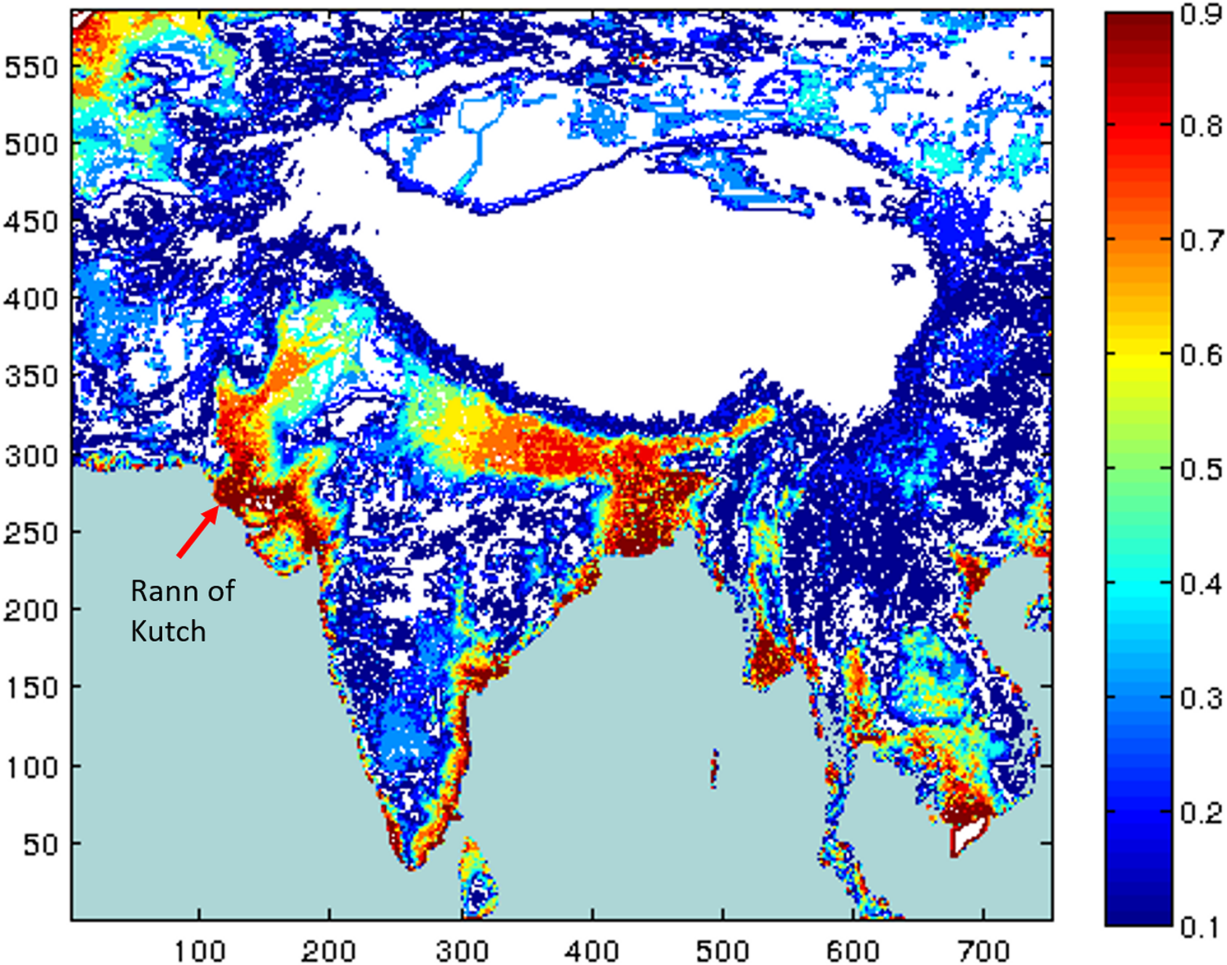
Habitability. Habitability of locations in the landmass of the South Asian subcontinent. The red regions indicate highly habitable places; white zones denote regions that lie more than 4,000 meters above sea level and are, therefore, deemed uninhabitable. Note that the Rann of Kutch is considered highly habitable due to the fact that it is below sea level and our computer algorithm assumes that all regions below sea level have adequate potable water.

As already stated we assume that people would act on their preference for locations that lie within 10 km of a water resource after which the desirability of a grid would fall steeply. Similarly, we assume that people would not like a location on a slope: the sharper the slope, the less would be the location’s desirability. Lastly, we assume that people would have a preference for locations whose altitude is close to sea level. Based on these assumptions we can create a desirability map of any region of interest (Fig 3). As described above, we dynamically define the difference between immigrating and emigrating populations to determine the population within each grid.

We have utilized satellite maps of the surface of the earth (GLOBE 1 km Digital Elevation Model from NOAA–the National Oceanic and Atmospheric Administration—http://www.ngdc.noaa.gov/mgg/topo/globe.html) along with high accuracy Hydro-shed data (http://hydrosheds.cr.usgs.gov/dataavail.php) for mapping of water resources. These satellite databases are publicly accessible at the websites noted above.

### Comparison of simulation results with genetic information: Error considerations

We compare the results of our simulations with available genetic data by focusing on those geographical regions in the subcontinent where different population groups are shown by our simulations to interact with each other 10,000 years after the commencement of the diffusion process. Potential errors in making such comparison are dependent on the following considerations. After only 10,000 years, no single population group is expected to dominate; neither does any population group reach saturation in a given geographical location. Consequently, all locations that we focus on are expected to display a mix of different genetic groups and saturation effects are unlikely to cause errors in our comparison. Moreover, saturation by a single dominant group is also made less likely by the endogamy that is common in these regions [5]. It is pertinent to note here that if population samples are taken further away from the regions where different population groups merge (Central India, in our case), saturation effects would be expected to begin manifesting themselves as one or more distinct genetic groups dominate; results of genetic mapping, indeed, shows this to be the case [3].

Errors can also arise in determining the precise geographical locations of the population groups that we focus on in making our comparisons. We convert the locations of the regions from which the genetic information is available into latitude and longitude. Taking into consideration the uncertainty associated with identification of the geographical locations on genetic maps, we estimate a half-degree error. The location of population merger of the northern group in our simulation (starting at Balkh in present-day Afghanistan) with the southern group (starting either at Hyderabad or Northern Orissa), we have made use of data from the corresponding coordinates of the concerned tribes and have determined a “goodness of fit” of the relative ancestral proportions from genetic data and from our computer simulation by the reduced chi-square test.

## Results and discussion

### Diffusion

We simulate the prehistoric movement of people in the subcontinent, starting notionally at 10,000 years before the present era. We select four geographical locations that seem to be most suitable for initial habitation based on evidence of Palaeolithic settlements [32]. ANI are believed, on the basis of genetic and archaeological information [14, 32], to have initially entered the subcontinent from Balkh [11] which is located west of the Khyber Pass. In the case of the ASI we choose a location within the Deccan plateau. Korisettar [32] has identified core areas of paleolithic occupation in the subcontinent which are consistent with the model proposed by Reich and coworkers [5] wherein most extant populations of India result from admixture between ANI and ASI [5, 13]. Other lines of evidence, including widespread rice cultivation in East and Northeast India [33, 34], abundance of ATB and AAA language speakers [12, 35], and other archeological, anthropometric [36] and genetic studies [37, 38] support the notion of waves of migration through northeast corridor in the subcontinent. In the southern region the Cuddapah basin is identified as a primary location of habitation [32]. Within this region we have specifically selected Hyderabad as the initial location for ASI diffusion. However, since the Deccan Plateau is relatively featureless, it might be expected to facilitate rapid expansion of population. Indeed, we have carried out tests by conducting simulations using different initial locations; results have shown that varying the exact initial location does not significantly affect the diffusion dynamics in this region. In this context it is important to note that relative insensitivity to the precise initial location of populations was also checked by us vis-a-vis ANI ingress: we substituted Balkh by locations corresponding to Mohenjodaro and Harappa and the results of our diffusion process was essentially unaltered over the 10,000 year period.

**Fig 4:**
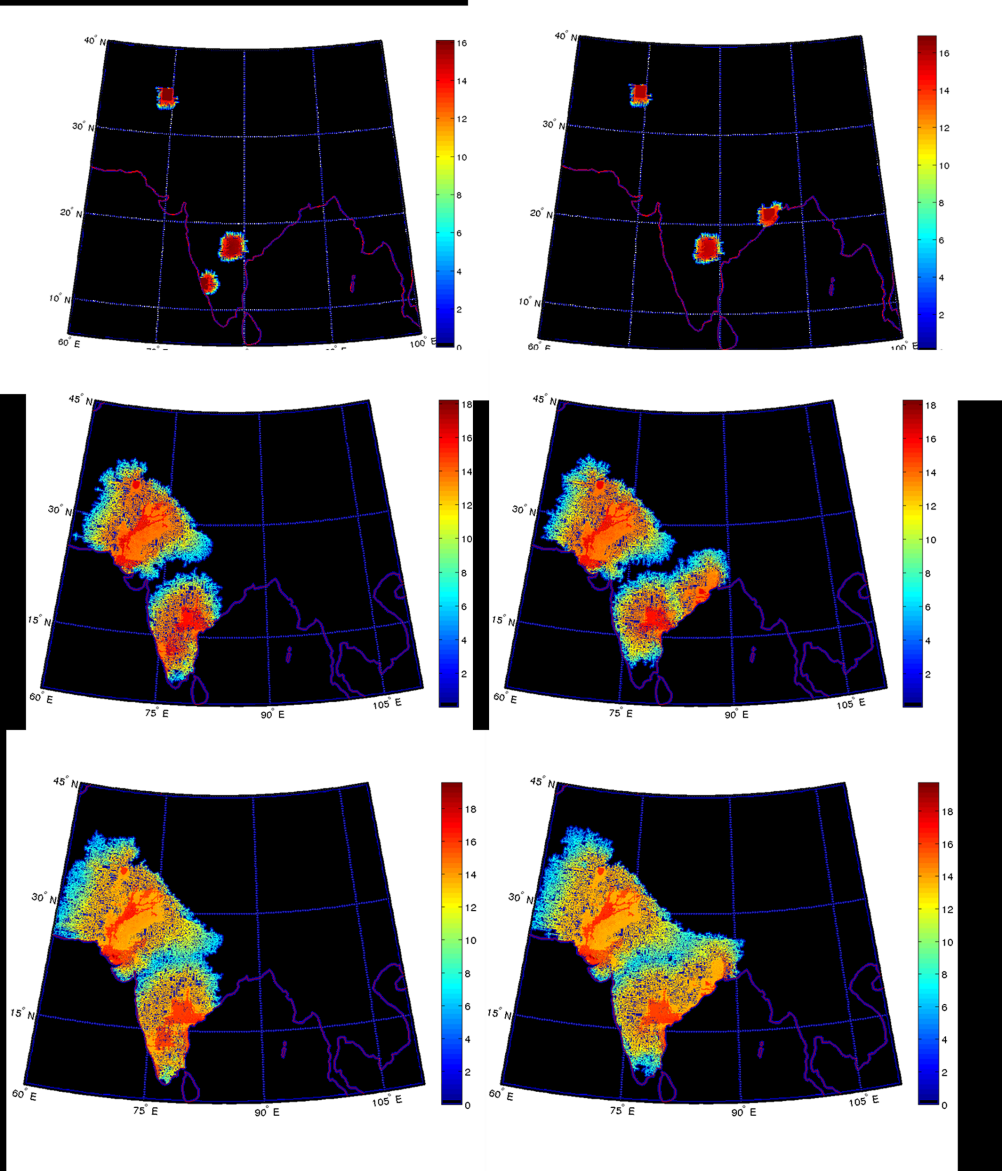
Computer simulations of population dynamics. Computer simulations of the population dynamics of the South Asian subcontinent using two different initial geographical locations (panels a and d). Panels b and e represent progress of the simulation after 500 steps (~4,000 years), and panels c and f after 800 steps (~6,400 years). Note the high degree of similarity in the “final” population maps in panels c and f.

The entry point of the AAA into the mainland from the coast is not clear but two distinct regions may be identified: these are locations at which the mountain ranges of the Western Ghats and the Eastern plateau break—corresponding to present day Goa and the Bastar region of northern Orissa [32]. We have used both of these starting points of AAA movement (Fig 4A and 4D) in different sets of computer simulations and the principle difference between the evolutions in these two scenarios is depicted in Fig 4. The figures in the left column (a-c) have their starting point in Goa, Hyderabad and Balkh while figures in the right column (d-f) have their starting point in northern Orissa, Hyderabad and Balkh. The first row of figures (a,d) show the initial points of simulation; the middle (b,e) and lowest panels (c,f) show, respectively, the progress of the simulation after 500 steps (~4,000 years) and 800 steps (~6,400 years).

As can be seen from Fig 4, when the AAA population enters the Deccan Plateau from Goa, it appears to merge with the ASI population in southern Karnataka. This merged group then goes on to meet the ANI population in central India. As a result one may not expect any population group in Eastern India with significant AAA and ANI genes (Fig 4C). This is not in agreement with observations that have been reported of significant AAA genes in Eastern India [12]. We, therefore, pursue an alternative scenario (Fig 4D) where the AAA population is assumed to have entered through northern Orissa.

It is pertinent to draw attention to the fact that results of our simulations shown in Fig 4C and 4F show clear evidence of the tributaries of the Indus river in the north western part of the subcontinent, thus establishing in visual fashion the obvious connection between population density and the desirability of living in close proximity to water resources.

The results shown in Fig 4 provide glimpses into how initial populations diffuse into new territories. Before discussing the information shown in this figure, it is worth noting that the peopling of India continues to be a contentious area of research and discourse [14]. Although there appears to be archaeological evidence that is suggestive of modern human habitation in the Arabian peninsula from about 120,000 to 75,000 years ago, in the case of the Indian subcontinent there is scarce archaeological data to suggest a modern human presence before about 50,000 years ago [18]. In our present studies we have taken cognizance of populations existing in the subcontinent at 30,000 years before the present; the entry routes of the major population groups at this time are shown in Fig 1 and they, indeed, serve as the seed populations for our diffusion-based simulations. Most importantly in the context of the results we present in this study, these results clearly show that the population dynamics over a course of 4,000 years or so do not seem to be too sensitive on the exact geographical location of the initial populations that we use in our computer simulations. Although this constitutes an important, positive facet of our method, we acknowledge that verifying the validity and accuracy of our results would have been difficult to establish until very recently. Availability of large-scale genetic data from geographically dispersed populations in recent times has opened new vistas for us to evaluate the results of our diffusion technique of studying population dynamics. We, therefore, use the recent results of genetic studies to evaluate the findings of our computer simulations in those geographical regions where different population groups merge with each other.

### Genetic studies on the peopling of India

Genetic analyses have, thus far, offered indications that Indian ethnic populations, when grouped as tribal or non-tribal or by geographic habitats, or linguistically, originate from a mix of 2 or 4 or 5 ancestral populations [5, 14, 39]. In addition to the original African ancestry, there seems to be a significant contribution to the Indian gene pool from West and Central Asia, as has been demonstrated by a number of studies [40–42]. More recent work has hinted at an admixture of two distinct ancestral contributions to the majority of the population—the ANI and ASI [5, 13, 43] and, most recently, Basu et al. [13] have reported four distinct ancestries in mainland India—the Ancestral Austro-Asiatic and Ancestral Tibeto-Burman in addition to the earlier ANI and ASI, and a separate Andamanese in the people of the Andaman and Nicobar Islands. Earlier genetic studies based on combinations of Y-chromosomal, X-chromosomal, autosomal SNP or mitochondrial DNA (mtDNA) analysis [39, 41, 44–48] yielded conflicting inferences. Polymorphisms in mtDNA and the non-recombining portions of the Y chromosome (NRY) have favoured spread through a single southern route [40], probably about 65 kya. The Asia-specific M and N haplogroups, which are derivatives of the L3 lineage found only in Africa [49], are found in high frequency in India [41]. The M2 haplogroup [41] and the Y-lineage M95-O2a haplogroup [48] are particularly frequent in Austro-Asiatic tribal populations and Dravidian-speaking populations of southern India. The latter places the origin of the AAA in Southeast Asia [2]. The mtDNA lineage U, which probably originated in Central Asia, has a high frequency in India—predominantly among high-ranking northern Indian castes but lower frequency in tribal populations. Thus, the view of a majority Central and West Eurasian genetic contribution has been modulated, and would likely have occurred after the early southern settlement [50]. More recent studies have relied on whole-genome analysis–the gold standard. These have provided further insights but fail to reach a consensus as well [5, 12–14, 43], probably due to differences or limitations of population sampling, methodology or statistical analysis.

The two recent whole genome studies of Moorjani et al. [13] and Basu et al. [14] based their population sampling upon regional linguistic distribution. Moorjani et al. [13] reported that an admixture of ANI and ASI ancestral populations in India that occurred about 4,200–1,900 years ago. The admixture was seen as a north-to-south clinal arrangement of individuals, with a decrease in the proportion of the ANI going southwards. They, however, excluded the TB and AA-speakers who were “off-cline” from their analysis. Basu et al. [14] performed a systematic analysis of genome-wide data on 367 unrelated individuals from 18 mainland and 2 island (Andaman and Nicobar) tribes, representing geographic, linguistic and ethnic diversities. Comparisons of the data of these populations were performed with others from the Human Genome Diversity Panel. They found varying proportions of four major ancestries in mainland India- corresponding to the four major linguistic groups. They also found that the AAA and ASI were the earliest settlers in India, possibly arriving by the southern coastal route from Africa. Differentiation between the AAA and ASI probably took place after their arrival into India. The ANI and ATB were, probably, rooted in the Central and east Asian populations who entered India by the north-western and north-eastern corridors. The ancestral populations occupied separate geographical habitats earlier, although there was some degree of admixture and some geographical displacements. Evidence for this was provided by detection of multiple ancestry components in extant populations. The Andamanese had a fifth, distinct ancestry with links to Oceanic (Pacific Islander) populations. Haplotype analysis also revealed widespread admixture of extant mainland populations, irrespective of ancestry, until about 70 generations ago (1 generation ~ 22.5 years [14])–that is, about 1,600 years ago—after which endogamy became prevalent especially among the upper castes and the Indo-European speakers. Tribal groups appeared to have followed a similar pattern, albeit less uniformly.

**Fig 5:**
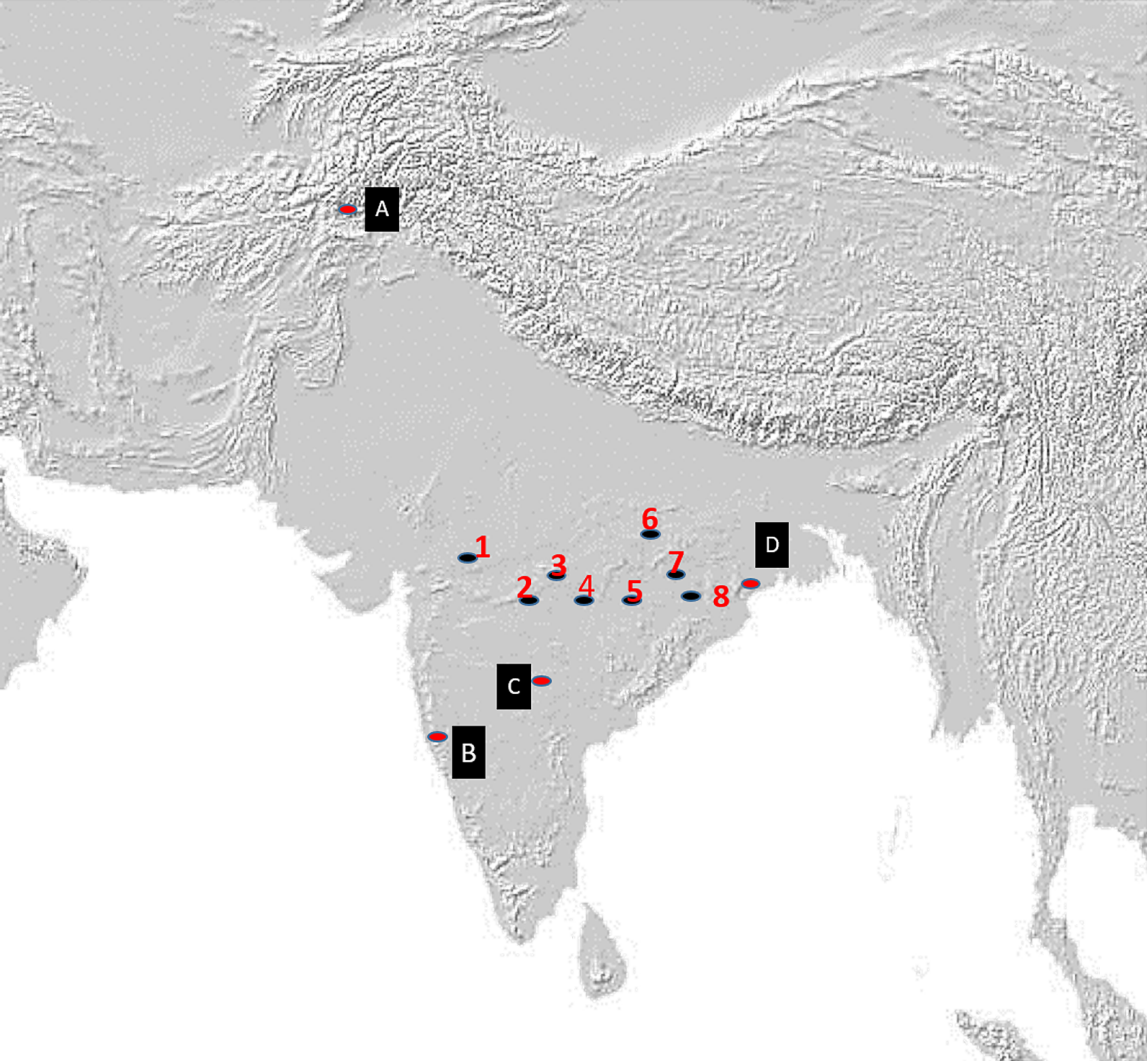
Location of some central Indian tribes. Location of tribes of Central India selected for genetic comparison (13, 24) and the simulation. A-D: Initial locations of the start of our computer simulation. A is located at present-day Balkh in present-day Afghanistan, B is at Goa, C is at Hyderabad, and D is located in Bastar in present-day Orissa. Location of the eight tribes used in the present study are marked using numerals 1 to 8 as follows: 1- Bhil, 2- Sahariya, 3- Gond, 4- Ho, 5- Santal, 6- Bharia, 7- Korwa, 8- Birhor.

While Basu et al. [14] and Moorjani et al. [13] report their own measurements along with those carried out by other researchers, we restrict our study only to the tribes studied directly by Basu et al. [14] and Sharma et al. [48] since these are the only tribes in Central India, our region of interest where different population groups are seen to merge in the course of our simulations. Tribal groups in India are representatives of autochthonous populations due to their relative social isolation. Genetic studies have focussed on tribes of the central Indian region due to their strategic geographical location along the merger of ancestral populations, as evidenced by the existence in close proximity of at least three of the major ancestral language families—the ANI, ASI and AAA. Genetic ancestries of some tribal populations of the central Indian state of Madhya Pradesh, such as the Bhil, Bharia and Sahariya [48] and the Gond, Ho, Santal, Korwa and Birhor [14] (Fig 5), have been reported and they provide an opportunity for comparison with our simulation results. Basu et al. [14] and Sharma et al. [48] point to the location of the region from which they took the data. We convert it to latitude and longitude. However, since the location dots are large, a half degree error is expected. At the region of population merger of the northern group in our simulation starting at Balkh with the southern group (starting point Hyderabad or Northern Orissa), we use data from the corresponding coordinates of these tribes and determine a “goodness of fit” of the relative ancestral proportions from genetic data and from our computer simulation by the reduced chi-square test (Table 1). Since the contribution of the fourth major language family—the ATB—in this region is negligible, we exclude it from the analysis. As is evident from the table, the goodness-of-fit values for the proportions of ANI (0.73), ASI (0.86) and AAA (0.77) of the selected tribal groups indicate a fairly good correlation between the genetic data and our simulation as also depicted graphically in Fig 5.

**Table 1:** Location and genetic contributions from the three major ancestral groups to the sampled population of select tribes of central India correlated with the simulation data. The numbers in the columns marked ANI, ASI, and AAA correspond to Ancestral North Indian, Ancestral South Indian, and Ancestral Austro-Asiatic, respectively. Genetic contributions are taken from the studies of Sharma et al. [48] and Basu et al. [14]. Statistical correlation has been calculated by the reduced chi-square test, and goodness of fit between the simulation data at the corresponding longitude and the genetic fraction of the different tribes is indicated in the last row.

Tribe	Longitude	Latitude	ANI	ASI	AAA
Bhil	75.5	22.5	0.36	0.466	0.1
Sahariya	78	21	0.22	0.22	0.54
Gond	79	22	0.3697	0.193	0.3756
Ho	80	21	0.0475	0.1705	0.7116
Santal	82	21	0.0347	0.1933	0.6398
Bharia	82.5	23.5	0.08	0.079	0.55
Korwa	83.5	22	0.0181	0.0471	0.9091
Birhor	84	21	0.0082	0.0054	0.9864
Fit to data			0.73	0.86	0.77

**Fig 6:**
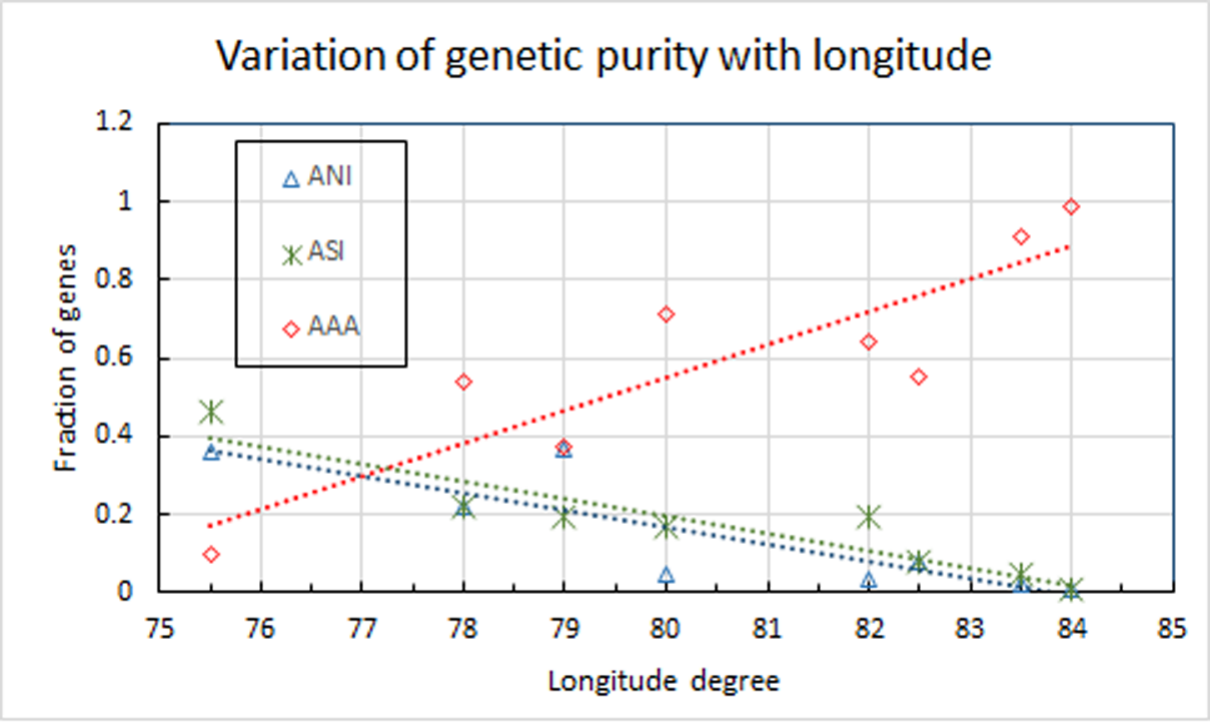
Latitude-dependence of relative gene fractions. Plot of longitude pertaining to the genetic data for different tribes as a function of the relative fraction of ANI, ASI, and AAA genes amongst them. The lines joining the points are the best fits. Note that as one travels from West to East, the fraction of ANI and ASI genes decreases monotonically. On the other hand, the fraction of AAA genes increases. This observation is consistent with the results of our simulations (Fig 4F).

We present data in Table 1 (as well as in Fig 6) for the latitude and longitude of the locations of each tribe whose genetic composition with regard to contributions from the major ancestral groups have been provided by Sharma et al. [48] and Basu et al. [14]. The results of our simulations (Fig 4F) seem to offer suggest that the proportions of genetic contributions in various tribes should have a longitude dependence for roughly similar values of latitude. The data selected by us meets this requirement. We have then plotted the fraction of ANI, ASI and AAA genes as a function of longitude for each of the tribes which are considered to have interacted with other population groups and became isolated only about 70 generations ago (~1,600 YBP). ATB provides insignificant contribution in this region and have, therefore, been excluded. The corresponding goodness of fit (chi-square) is presented in the last row in Table 1. We find that the fraction of AAA genes appears to increase steeply at higher longitudes in the East while the fraction of ANI and ASI genes seems to decrease significantly towards the easterly direction. This observation is consistent with the results of our simulations (Fig 4F).

As to comparisons between our simulation results and genetics information from other parts of the subcontinent, we are not able to come up with anything other than what might be considered to be obvious. For instance, we have followed the ANI population distribution as one goes from the north to the south: we find that the ANI component reduces, as would be expected from genetic data [5, 14]. Similarly, the ASI component descreases as one goes from south to north. Our present model has the limitation that more meaningful comparsions are not possible other than in regions where population merger occurs.

## Concluding remarks

We have simulated, over a period of 10,000 years, the movement of the three ancestral groups—AAA, ANI and ASI. Our simulation starts at a time point corresponding to 10,000 YBP; 500 steps and 800 steps in our simulations correspond to 4000 and 6400 years, respectively, after the start of our simulations. Hence, our primary simulation with 500 steps (whose results are depicted in Fig 4) ends at a time point corresponding to 6000 YBP. Correspondingly, the 800-step simulation ends at a time point corresponding 3600 YBP. The time period covered by our simulations takes cognizance of the seed population that resulted from the demographic expansion that occurred in the Indian subcontinent around 30,000 YBP [51].

**Fig 7:**
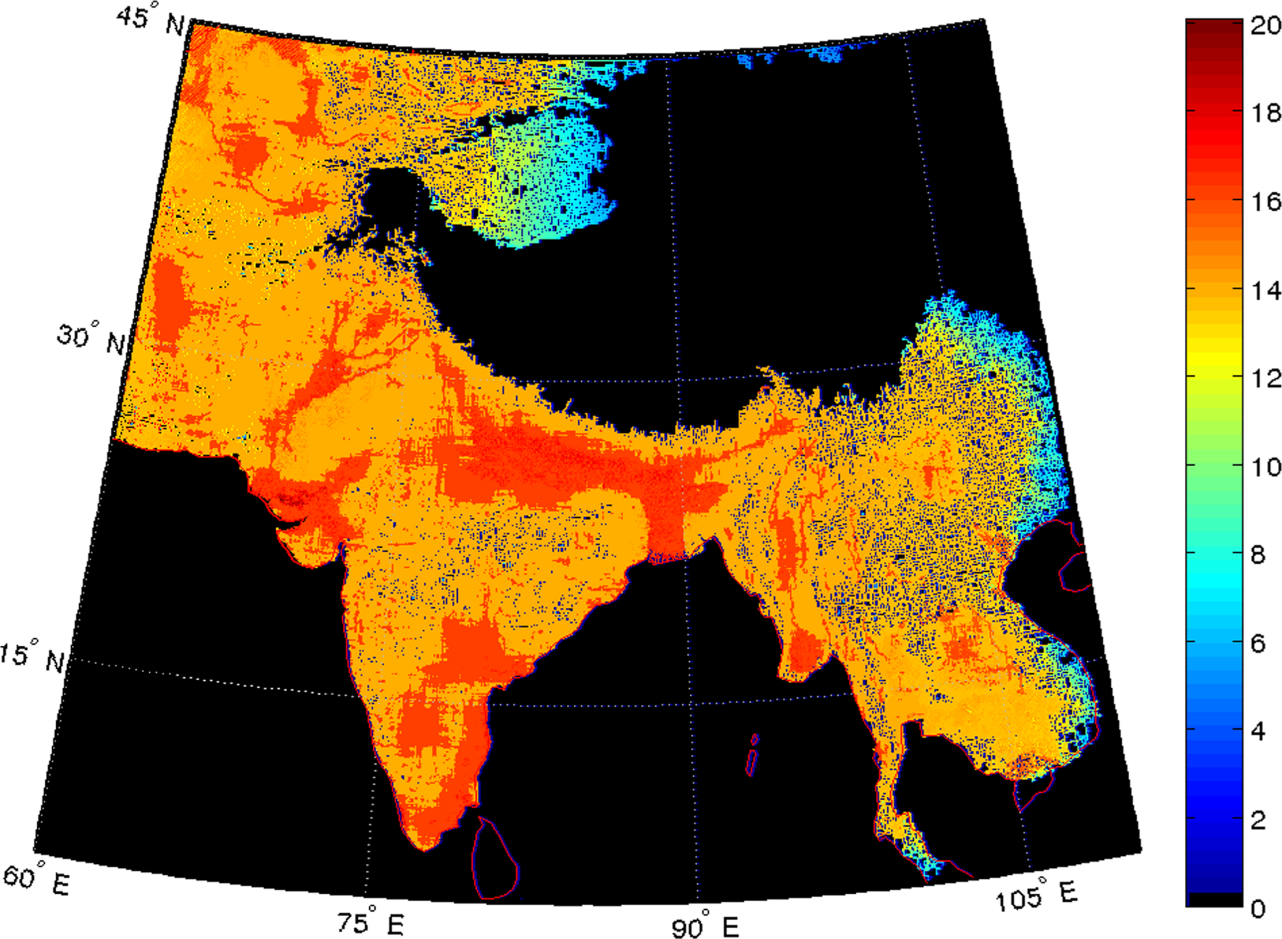
Extended simulations. Temporal extension of the simulation from 800 steps (Fig 3F) to 4,000 steps. The population is seen to skirt around the Tibetan plateau and spread into eastern China. The simulation also suggests that the population entering the Taklamakan Desert would have had a single entry point from the east.

The results of our simulations seem to suggest that the scenario that best fits the available genetic data involves entry of AAA into the subcontinent from the Easterly direction. This seems to agree with the fact that the Austro-Asiatic tribes (such as Ho, Santal, Korwa, Birhor) are largely seen in this region, extending to eastern Maharashtra. Our simulations indicate that the initial populations start to coalesce around 4,000 YBP before the commencement of a period of relative geographical isolation of each population group. The period during which coalescence of populations occurs appears consistent with the established timeline associated with the Harappan civilization [52] and, also, with the genetic admixing that recent genetic mapping data reveal. However, the simulations that we have performed permit us to extend our study even further—they also provide a hitherto unavailable timeline of the movement of people. Apart from the broad features of merger of populations, we also notice that several regions within the subcontinent are mountainous. As a result, the movement of the population in such locations seems to take place in temporal spurts, with small groups penetrating through an occasional gap in the mountains and then spreading over a large open valley. In such regions the small groups along with enforced endogamy ensures that the local group tends to preserve the random genetic signals of their precursors even though they provide no significant biological advantage. To illustrate this, in Fig 7 we have extended the simulation to 4,000 steps. As a result of this, the population now extends towards China, bypassing the high Tibetan plateau in the north. In the east, the simulation shows the spread of the population into East Asia and South China. Particularly interesting is the fact that the simulation suggests that the population entering the Taklamakan Desert may have a single entry point from the east. The manner in which the delta of the Irrawaddy river in the south is populated is also suggestive of this group going to the Andaman and Nicobar islands, as is, indeed, suggested by genetic data [12].

It is of interest to compare the results shown in Fig 7 with present-day population density maps. The latter are publicly accessible from the National Centre for Geographic Information (http://www.ncgia.ucsb.edu/pubs/gdp/pop.html) and the website http://www.globalwaterforum.org/wp-content/uploads/2013/08/Figure-1.png. The broad population contours obtained from our simulations appear to be broadly in consonance with present-day populations densities in the sub-continent. However, it is important to note the population dynamics is not expected to be linear in the latter periods of our simulations due to a variety of factors, such as improvement in transportation technologies and higher population pressures. Such factors will contribute to increasing the rate of population diffusion beyond what is taken account of in Eq 1.

The results of the present studies lead us to conclude that simulating the movement of the prehistoric peoples of India on the basis of a diffusion model yields results that are consistent with the genetic profile of the tribal populations of India in regions where populations mergers have occurred. Our methodology may, therefore, find utility in a variety of other purposes, such as identifying the time at which human population originated in a given geographical location.

In this study we have also been able to establish that our diffusion method appears to accommodate well a surprising degree of latitude as far as the placement of initial starting points is concerned. For instance, our simulation results indicate that the exact location of the point of origin in south India does not appear to be critical to the profile of the spread of the ASI population in India. Similarly, the simulation seems to be not very sensitive to initial ANI population locations. However, the entry of the AAA into the subcontinent would be most efficient through the points of break in the Western Ghats and the Eastern Ghats. However, if we assume that the entry occurred from Goa (Fig 4A and 4C) the genetic profiles of the Central Indian tribes would be far different from what has hitherto been reported in the literature. The fit is seen to be much better with our simulation if we assume that the AAA entered through Northern Orissa, thus resolving a hitherto-contentious issue.

The methodology that we have reported here may help establish a timeline for the movement of prehistoric people, to help define subtle aspects of how different populations merged, and provide a different insight into the long-term temporal and spatial evolution of prehistoric populations. Our simulations may also help identify the most favourable locations for the early populations to have settled down in. We anticipate that our simulation method might be of general and efficient utility in a variety of population dynamics studies and may provide a quick, fairly reliable method that may, perhaps, obviate the need for extensive genetic profiling and analysis.

## References

[1] Barceló JA, Castillo FD, editors. Simulating Prehistoric and Ancient Worlds, Springer; 2016.

[2] Vahia MN, Ladiwala U, Mahathe P, Mathur D., Population Dynamics of Early Human Migration in Britain. PLoS ONE. 2016; 11(5): e0154641 doi: 10.1371/journal.pone.0154641 27148959 10.1371/journal.pone.0154641 PMC4858239

[3] Tamang R, Thangaraj K. Genomic view on the peopling of India. Investig. Genet. 2012; 3: 20 doi: 10.1186/2041-2223-3-20 23020857 10.1186/2041-2223-3-20 PMC3514343

[4] Normile D. SNP study supports southern migration route to Asia. Science. 2009; 326: 1470–1476. doi: 10.1126/science.326.5959.1470 20007876 10.1126/science.326.5959.1470

[5] Reich D, Thangaraj K, Patterson N, Price AL, Singh L. Reconstructing Indian population history. Nature. 2009; 461: 489–494. doi: 10.1038/nature08365 19779445 10.1038/nature08365 PMC2842210

[6] Chakravarti A. Human genetics: Tracing India's invisible threads. Nature. 2009; 461: 487–488. doi: 10.1038/461487a 19779444 10.1038/461487a

[7] Culotta E, Gibbons A. Aborigines and Eurasians rode one migration wave. Science. 2016; 353: 1352–1353. doi: 10.1126/science.353.6306.1352 27708018 10.1126/science.353.6306.1352

[8] Alves I, Šrámková Hanulová A, Foll M, Excoffier L. Genomic Data Reveal a Complex Making of Humans. PLoS Genetics. 2012; 8: e1002837 doi: 10.1371/journal.pgen.1002837 22829785 10.1371/journal.pgen.1002837 PMC3400556

[9] Finlayson C. Biogeography and evolution of the genus Homo. Trends Ecol Evol. 2005; 20: 457–63. doi: 10.1016/j.tree.2005.05.019 16701417 10.1016/j.tree.2005.05.019

[10] Dennell R, Petraglia MD. The dispersal of Homo sapiens across southern Asia: how early, how often, how complex? Quaternary Science Reviews. 2012; 47: 15–22.

[11] Possehl GL. Thoughts on the evolution and history of human populations in South Asia In: Petraglia MD, Allchin B, editors. The Evolution and History of Human Populations in South Asia. Dordrecht: Springer; 2007 pp. 447–459.

[12] Chaubey G, Metspalu M, Choi Y, Mägi R, Romero IG, Soares P, et al Population genetic structure in Indian Austroasiatic speakers: The role of landscape barriers and sex-specific admixture. Mol. Biol. Evol. 2011; 28: 1013–1024. doi: 10.1093/molbev/msq288 20978040 10.1093/molbev/msq288 PMC3355372

[13] Moorjani P, Thangaraj K, Patterson N, Lipson M, Loh PR, Govindaraj P, et al Genetic evidence for recent population mixture in India. Am. J. Human Genet. 2013; 93: 422–438. 23932107 10.1016/j.ajhg.2013.07.006 PMC3769933

[14] Basu A, Sarkar-Roy N, Majumder PP. Genomic reconstruction of the history of extant populations of India reveals five distinct ancestral components and a complex structure. Proc. Natl. Acad. Sci. 2016; 113, 1594–1599. doi: 10.1073/pnas.1513197113 26811443 10.1073/pnas.1513197113 PMC4760789

[15] Allchin B, Allchin FR. The Rise of Civilization of India and Pakistan. Cambridge: Cambridge University Press; 1982.

[16] Morrison KD. Foragers and forager traders in South Asian World: Some thoughts from the last 10,000 years In: Petraglia MD, Allchin B, editors. The Evolution and History of Human Populations in in South Asia. Dordrecht: Springer; 2007 pp. 321–339.

[17] Paddayya K. The Acheulean of peninsular India with special reference to the Hungsi and Baichbal valleys of the lower Deccan In: Petraglia MD, Allchin B, editors. The Evolution and History of Human Populations in South Asia. Dordrecht: Springer; 2007 pp. 97–119.

[18] deMenocal PB and Stringer C. Human migration: Climate and the peopling of the world. Nature. 2016; 538: 49–50. doi: 10.1038/nature19471 27654915 10.1038/nature19471

[19] Timmermann A and Friedrich T. Late Pleistocene climate drivers of early human migration. Nature. 2016; 538: 92–95. doi: 10.1038/nature19365 27654920 10.1038/nature19365

[20] Ambrose SH. Late Pleistocene human population bottlenecks, volcanic winter, and differentiation of modern humans. J Hum Evol. 1998; 34: 623–651. doi: 10.1006/jhev.1998.0219 9650103 10.1006/jhev.1998.0219

[21] Castañeda IS, Mulitza S, Schefuß E, Lopes dos Santos RA, Sinninghe Damsté JS, Schouten S. Wet phases in the Sahara/Sahel region and human migration patterns in North Africa. Proc Natl Acad Sci U S A. 2009; 106: 20159–20163. doi: 10.1073/pnas.0905771106 19910531 10.1073/pnas.0905771106 PMC2776605

[22] Eriksson A, Betti L, Friend AD, Lycett SJ, Singarayer JS, von Cramon-Taubadel N, et al Late Pleistocene climate change and the global expansion of anatomically modern humans. Proc Natl Acad Sci U S A. 2012; 109: 16089–16094. doi: 10.1073/pnas.1209494109 22988099 10.1073/pnas.1209494109 PMC3479575

[23] Osborne AH, Vance D, Rohling EJ, Barton N, Rogerson M, Fello N. A humid corridor across the Sahara for the migration of early modern humans out of Africa 120,000 years ago. Proc Natl Acad Sci USA. 2008; 105: 16444–16447. doi: 10.1073/pnas.0804472105 18936490 10.1073/pnas.0804472105 PMC2575439

[24] Perry CA, Hsu KJ. Geophysical, archaeological, and historical evidence support a solar-output model for climate change. PNAS. 2000; 97: 12433–12438. doi: 10.1073/pnas.230423297 11050181 10.1073/pnas.230423297 PMC18780

[25] Hong YT, Hong B, Lin QH, Zhu YX, Shibata Y, Hirota M, et al Correlation between Indian Ocean summer monsoon and North Atlantic climate during the Holocene. Earth Planet. Sci. Lett. 2003; 211: 371–380.

[26] Field JS, Petraglia MD, Lahr MM. The southern dispersal hypothesis and the South Asian archaeological record: Examination of dispersal routes through GIS analysis. J. Anthropo. Archaeol. 2007; 26: 88–108.

[27] Wagle BG, Vora KH, Karisiddaiah SM, Veerayya M, Almeida F. Holocene submarine terraces on the western continental shelf of India; Implications for sea-level changes. Marine Geol. 1994; 117: 207–225.

[28] Hastings DA, Dunbar P. Development & Assessment of the Global Land One-km Base Elevation Digital Elevation Model (GLOBE). ISPRS Archives.1998; 32: 218–221.

[29] Lehner B, Verdin K, Jarvis A. New global hydrography derived from spaceborne elevation data. Eos, Trans. Am. Geophys. Union. 2008; 89: 93–94.

[30] Cohen JE and Small C. Hypsographic demography: The distribution of human population, by altitude. Proc. Natl. Acad. Sci. USA. 1998; 95: 14009–14014. 9826643 10.1073/pnas.95.24.14009 PMC24316

[31] Kummu M, de Moel H, Ward PJ, Varis O. How Close Do We Live to Water? A Global Analysis of Population Distance to Freshwater Bodies. PLoS ONE. 2011; 6: e20578 doi: 10.1371/journal.pone.0020578 21687675 10.1371/journal.pone.0020578 PMC3110782

[32] Korisettar R. Toward developing a basin model for Paleolithic settlement of the Indian subcontinent: Geodynamics, monsoon dynamics, habitat diversity and dispersal routes In: Petraglia MD, Allchin B, editors. The Evolution and History of Human Populations in South Asia. Dordrecht: Springer; 2007 pp. 69–96.

[33] Diamond J, Bellwood P. Farmers and their languages: The first expansions. Science, 2003; 300: 597–603. doi: 10.1126/science.1078208 12714734 10.1126/science.1078208

[34] Kumar V, Reddy AN, Babu JP, Rao TN, Langstieh BT, Thangaraj K, et al Y-chromosome evidence suggests a common paternal heritage of Austro-Asiatic populations. BMC Evol. Biol. 2007; 7:47 doi: 10.1186/1471-2148-7-47 17389048 10.1186/1471-2148-7-47 PMC1851701

[35] Chaubey G, Metspalu M, Kivisild T, Villems R. Peopling of South Asia: Investigating the caste-tribe continuum in India. BioEssays. 2007 29:91–100. doi: 10.1002/bies.20525 17187379 10.1002/bies.20525

[36] Thapar R. Early India: From the Origins to AD 1300. Berkeley, CA: Univ of California Press; 2004.

[37] Basu A, Mukherjee N, Roy S, Sengupta S, Banerjee S, Chakraborty M, et al Ethnic India: A genomic view, with special reference to peopling and structure. Genome Res. 2003; 13: 2277–2290. doi: 10.1101/gr.1413403 14525929 10.1101/gr.1413403 PMC403703

[38] Abdulla MA, Ahmed I, Assawamakin A, Bhak J, Brahmachari SK, Calacal GC, et al; HUGO Pan-Asian SNP Consortium; Indian Genome Variation Consortium. Mapping human genetic diversity in Asia. Science. 2009 326:1541–1545. doi: 10.1126/science.1177074 20007900 10.1126/science.1177074

[39] Brahmachari SK, Majumder PP, Mukerji M, Habib S, Dash D, Ray K, et al Genetic landscape of the people of India: a canvas for disease gene exploration. J. Genet. 2008; 87: 3–20. 18560169 10.1007/s12041-008-0002-x

[40] Kivisild T, Rootsi S, Metspalu M, Mastana S, Kaldma K, Parik J, et al The genetic heritage of the earliest settlers persists both in Indian tribal and caste populations. Am. J. Human Genet. 2003; 72: 313–332. 12536373 10.1086/346068 PMC379225

[41] Sengupta S, Zhivotovsky LA, King R, Mehdi SQ, Edmonds CA, Chow CE, et al Polarity and temporality of high-resolution Y-chromosome distributions in India identify both indigenous and exogenous expansions and reveal minor genetic influence of Central Asian pastoralists. Am. J. Human Genet. 2006; 78: 202–221. 16400607 10.1086/499411 PMC1380230

[42] Sharma S, Rai E, Sharma P, Jena M, Singh S, Darvishi K, et al The Indian origin of paternal haplogroup R1a1* substantiates the autochthonous origin of Brahmins and the caste system. J. Human Genet. 2009; 54: 47–55. 19158816 10.1038/jhg.2008.2

[43] Metspalu M, Romero IG, Yunusbayev B, Chaubey G, Mallick CB, Hudjashov G, et al Shared and unique components of human population structure and genome-wide signals of positive selection in South Asia. Am. J. Human Genet. 2011; 89: 731–44. 22152676 10.1016/j.ajhg.2011.11.010 PMC3234374

[44] Kivisild T, Bamshad MJ, Kaldma K, Metspalu M, Metspalu E, Reidla M, et al Deep common ancestry of indian and western-Eurasian mitochondrial DNA lineages. Curr. Biol. 1999; 9: 1331–1334. 10574762 10.1016/s0960-9822(00)80057-3

[45] Metspalu M, Kivisild T, Metspalu E, Parik J, Hudjashov G, Kaldma K, et al Most of the extant mtDNA boundaries in South and Southwest Asia were likely shaped during the initial settlement of Eurasia by anatomically modern humans. BMC Genetics. 2004; 5: 26 doi: 10.1186/1471-2156-5-26 15339343 10.1186/1471-2156-5-26 PMC516768

[46] Sahoo S, Singh A, Himabindu G, Banerjee J, Sitalaximi T, Gaikwad S, et al A prehistory of Indian Y chromosomes: Evaluating demic diffusion scenarios. Proc. Natl. Acad. Sci. 2006; 103: 843–848. doi: 10.1073/pnas.0507714103 16415161 10.1073/pnas.0507714103 PMC1347984

[47] Thangaraj K, Chaubey G, Singh VK, Vanniarajan A, Thanseem I, Reddy AG, et al In situ origin of deep rooting lineages of mitochondrial Macrohaplogroup 'M' in India. BMC Genomics. 2006; 7: 151 doi: 10.1186/1471-2164-7-151 16776823 10.1186/1471-2164-7-151 PMC1534032

[48] Sharma G, Tamang R, Chaudhary R, Singh V K, Shah AM, Anugula S, et al Genetic Affinities of the Central Indian Tribal Populations. PLoS ONE. 2012; 7: e32546 doi: 10.1371/journal.pone.0032546 22393414 10.1371/journal.pone.0032546 PMC3290590

[49] Quintana-Murci L, Semino O, Bandelt HJ, Passarino G, McElreavey K, Santachiara-Benerecetti AS. Genetic evidence of an early exit of Homo sapiens sapiens from Africa through eastern Africa. Nat Genet. 1999; 23: 437–441. doi: 10.1038/70550 10581031 10.1038/70550

[50] Palanichamy MG, Sun C, Agrawal S, Bandelt H-J, Kong Q-P, Khan F, et al Phylogeny of Mitochondrial DNA Macrohaplogroup N in India, Based on Complete Sequencing: Implications for the Peopling of South Asia. Am. J. Human Genet. 2004; 75: 966–978. 15467980 10.1086/425871 PMC1182158

[51] Petraglia MD, Allchin B. Human evolution and culture change in the Indian subcontinent In: Petraglia MD, Allchin B, editors. The Evolution and History of Human Populations in in South Asia. Dordrecht: Springer; 2007 pp. 1–20.

[52] Gangal K, Vahia MN, Adhikari R. Spatio-temporal analysis of the Indus urbanization. Current Science. 2010; 98: 846–852.

